# Types of HPV Vaccine Misinformation Circulating on Twitter (X) That Parents Find Most Concerning: Insights From a Cross-Sectional Survey and Content Analysis

**DOI:** 10.2196/54657

**Published:** 2025-05-12

**Authors:** Jennifer C Morgan, Sarah Badlis, Katharine J Head, Gregory Zimet, Joseph N Cappella, Melanie L Kornides

**Affiliations:** 1 Department of Family and Community Health School of Nursing University of Pennsylvania Philadelphia, PA United States; 2 Communication Studies School of Liberal Arts Indiana University Indianapolis, IN United States; 3 Department of Pediatrics School of Medicine Indiana University Indianapolis, IN United States; 4 Annenberg School for Communication University of Pennsylvania Philadelphia, PA United States

**Keywords:** misinformation, social media, HPV vaccine, vaccine uptake, parental concern, vaccine misinformation, Twitter, cross-sectional survey, content analysis, Tweets, engagement

## Abstract

**Background:**

Parents frequently use social media as a source of information about the human papillomavirus (HPV) vaccine. Our previous work identified that, on Twitter (now X), almost 25% of tweets about the HPV vaccine contain misinformation, and these tweets receive higher audience engagement than accurate tweets. Exposure to misinformation can increase vaccine hesitancy, but the types of misinformation found on social media vary widely, and not all misinformation exposure influences vaccine attitudes and vaccine uptake. Despite the prevalence of misinformation and antivaccine information on social media, little work has assessed parents’ assessments of these posts.

**Objective:**

This study examines which types of misinformation on Twitter parents find the most concerning.

**Methods:**

In April 2022, we surveyed 263 US parents of children ages 7-10 years using a Qualtrics survey panel. They viewed a first round of 9 randomly selected tweets from a pool of 126 tweets circulating on Twitter that contained misinformation about the HPV vaccine. Then parents selected up to 3 that they found most concerning. The process was repeated once more with 9 selected from the pool of 117 messages not shown in the first round. Using this information, a concern score for each tweet was calculated based on the number of parents who viewed the tweet and selected it as concerning. In total, 2 researchers independently coded the misinformation tweets to identify rhetorical strategies used and health concerns mentioned. Multiple linear regression tested whether tweet content significantly predicted the concern score of the tweet.

**Results:**

Parental concern about the different misinformation tweets varied widely, with some misinformation being selected as most concerning just 2.8% of the time it was viewed and other misinformation being selected 79.5% of the time it was viewed. Multiple beta regression analyses found that misinformation tweets using negative emotional appeals (b=.79, *P*<.001), expressing pharmaceutical company skepticism (b=.36, *P*=.036), invoking governmental authority (b=.44, *P*=.02), and mentioning hospitalization (b=1.00, *P*=.003), paralysis (b=.54, *P*=.02), and infertility (b=.52, *P*=.04) significantly increased the percent of parents rating the misinformation tweets as most concerning.

**Conclusions:**

Misinformation about HPV vaccination is ubiquitous on social media, and it would be impossible to target and correct all of it. Counter-messaging campaigns and interventions to combat misinformation need to focus on the types of misinformation that concern parents and ultimately may impact vaccine uptake. Results from this study identify the misinformation content that parents find most concerning and provide a useful list of targets for researchers developing interventions to combat misinformation with the goal of increasing HPV vaccine uptake.

## Introduction

### Background

Approximately 13 million Americans are infected with the human papillomavirus (HPV) each year [1]. HPV is a group of more than 200 related viruses, many of which are spread through vaginal, anal, or oral sex [2]. Certain high-risk and persistent HPV infections can lead to developing HPV-related cancers, which include cervical, oropharyngeal, and anal cancers [2]. Every year in the United States, over 20,000 women and 14,000 men are diagnosed with cancers caused by HPV, and over 4000 women die from HPV-related cervical cancer [3].

HPV vaccination prevents new HPV infections and HPV-associated cancers [2]. Since the introduction of the HPV vaccine, infections with HPV have dropped 88% among teen girls and 81% among young adult women [4]. More than 135 million doses have been distributed in the United States [4], but HPV vaccine uptake is lower than coverage with most other routine vaccines [5]. In 2021, only 61.7% of teens were up to date with HPV vaccination, much lower than the goal of 80% set by Healthy People 2030 [6]. In comparison, levels of tetanus, diphtheria, and acellular pertussis (Tdap) and meningococcal ACWY vaccination coverage at 89.6% and 89.0% in 2021 exceeded public health goals [5].

### The Influence of Social Media and Misinformation on Vaccination

The current US social environment (eg, social norms about vaccination, provider recommendations, vaccine myths, and misinformation about vaccines) is influenced by social media. Nearly all parents of adolescents use social media, with 68% using social media for health information [7]. Parents seek health information from social media for convenience and to receive or provide information, support, advice, or validation for health decisions [8]. Antivaccine content is shared frequently and extensively on social media [9-11], and vaccine misinformation quickly disseminates via social media platforms, fueled by anecdotes, rumors, and trending hashtags, without editorial oversight [12]. In total, 41% of parents believe they are often or sometimes exposed to negative information about vaccination on social media [13]. Evidence suggests that exposure to vaccine misinformation on social media may directly influence vaccination opinions and contribute to downstream vaccine hesitancy [12,14-16].

Motivation to vaccinate refers to the intersecting constructs of intention, willingness, acceptability, hesitancy toward vaccines, and the social environment [17]. Motivation to vaccinate is one factor contributing to relatively low HPV vaccine uptake in the United States [17]. Parental vaccine hesitancy, “a state of indecisiveness regarding a vaccination decision, independently of behaviour” [18], is associated with significantly lower coverage for HPV vaccination among adolescents [19]. One study, using a national US sample, found that parental hesitancy about HPV vaccination among parents of adolescents 11-17 years of age is prevalent (23%) and has a stronger influence on vaccine uptake than practical barriers such as cost or access [20].

### HPV Vaccine Content on Social Media

Representation of the HPV vaccine on Facebook was negative within the first 10 years of HPV vaccine availability [21]. One study found that about a quarter of HPV-related content on Twitter contained misinformation about the HPV vaccine. HPV vaccine misinformation posts had a higher average “like” count than pro-vaccine posts, making them more likely to be seen by broader audiences [22]. Viewing pro-HPV vaccine content on social media ironically exposes parents to antivaccine rhetoric, as pro-HPV vaccine content attracts antivaccine responses [23].

The most common types of negative HPV vaccine posts on social media include inaccurate claims, conspiracy theories, concerns about vaccine safety and personal freedoms, and lack of vaccine efficacy; all are forms of disinformation or misinformation [10]. Currently, many US parents have expressed doubts about the benefits of the HPV vaccine and do not believe that the vaccine is effective at preventing HPV infection or protecting against HPV-related cancers [20]. Parents express concerns about HPV vaccine safety, the novelty of the vaccine, and mistrust of information about the vaccine received from their adolescent’s health care provider [20]. These concerns about vaccines are being raised and amplified by health misinformation on social media, with the HPV vaccine being the most impacted [24]. Parents of children who recalled seeing information about HPV and the HPV vaccine on social media were significantly more likely to perceive that the HPV vaccine could be lethal for their children, compared to parents who had reported not seeing information on social media [25].

The content of misinformation messages on social media ranges widely and includes several different persuasive strategies. Our recent Twitter study, conducted prior to the platform changing its name to X, found that the top 3 categories of misinformation about HPV vaccination were negative health effects, mandatory vaccination, and ineffectiveness [26]. Among adverse health–related misinformation tweets, non-specific health harm was the most common topic [26]. In the HPV antivaccine network, the main traits of social media misinformation messages included mentioning #Gardasil, claiming to reveal a lie, conspiracy theories, and risk of vaccine injury [22].

Previous research has documented the amount of misinformation about the HPV vaccine on social media, the most prevalent misinformation, and it has documented the ways that vaccine misinformation impacts vaccination attitudes and behavior. Given the high prevalence of misinformation on social media, knowing which types of misinformation parents find most concerning provides critical information for directing resources into addressing the misinformation that worries the most parents when they encounter it. In this study, we sought to identify the content and rhetorical strategies of misinformation tweets that were most concerning to parents of 7- to 10-year-olds.

## Methods

### Participants

In April 2022, a total of 263 US caregivers completed a web-based survey about HPV vaccine misinformation that was circulating on Twitter. Parents and legal guardians were recruited from Qualtrics Panels and were eligible if their oldest child was between 7 and 10 years old, they were the primary or shared medical decision maker for their child, and their child had not received the HPV vaccine. The survey took participants an average of 14 minutes to complete. The caregivers in the study were primarily mothers (n=198, 75%), 67% (n=199) were non-Hispanic White, and 54% (n=142) had a high school education or less. Their children had an average age of 8.96 (SD 0.98) years, and 49% (n=128) were male and 51% (n=135) female (Table 1).

**Table 1:** Participant characteristics (N=263). Demographic information from a cross-sectional Internet survey of 263 US caregivers.

Characteristics					Values
Age of the caregivers, mean (SD), range					38.68 (8.69), 21-76
Age of the children, mean (SD), range					8.96 (0.98), 7-10
**Caregivers’ characteristics,** **n (%)**					
	**Number of children**				
		One child	120 (46)		
		Two or more children	143 (54)		
	**Relationship with the children**				
		Mother	198 (75)		
		Father	49 (19)		
		Other legal guardian	16 (6)		
	**Race**				
		White	199 (76)		
		Black or African American	46 (17)		
		Asian, American Indian, Alaska Native, or multiracial	18 (7)		
	Hispanic ethnicity			33 (13)	
	**Highest education**				
		High school or less	142 (54)		
**Children’s characteristics** **,** **n (%)**					
	**Gender of the children**				
		Male	128 (49)		
		Female	135 (51)		
	**Race of the children**				
		White	181 (69)		
		Black or African American	48 (18)		
		Asian, American Indian or Alaska Native, or multiracial	34 (13)		
	Hispanic children			48 (18)	

### Stimuli

The stimuli in this study consisted of 126 images of real tweets that contained misinformation about the HPV vaccine. These tweets were part of a larger collection from our previous study, a retrospective content analysis conducted from December 2019 to April 2020 [26]. All English language tweets associated with #HPV posted during this time period were included in the study. For this study, we used the 126 tweets that were identified by human coders as raising health-related harm or injury concerns about the HPV vaccine. This category was associated with significantly higher levels of audience engagement (a sum of “like” count, retweet count, and reply count) in our previous study, which may suggest that viewers found this information particularly concerning. Tweets containing entirely duplicate information were removed. Tweets that contained partially duplicate information along with unique information were included. For example, in a case where a tweet was sentence A and another that was sentence A followed by sentence B, both tweets were retained. The stimuli retained the text of the original tweet including hashtags, @usernames and links in the body of the tweet. We disabled the hyperlink capabilities to prevent participants from leaving the survey. We redacted the name, username (ie, Twitter handle), and engagement metrics (shares, likes, and retweets) to control for any confounding influence of social norms or popularity that the engagement metrics might convey. Further information on the methods for tweet collection and identification of misinformation can be found in our prior study [26].

### Procedures

Caregivers answered screening and demographic questions about themselves and their oldest child (ages 7-10 years). Then they viewed a screen containing 9 randomly selected messages from the pool of 126 tweets and selected the 3 messages they found most concerning about receiving the HPV vaccine for their child. The process was repeated once more, drawing the second set of 9 messages from the remaining pool of 117 messages not shown in the first round. Each tweet had a 14% chance of being seen by a given participant and was viewed an average of 38 times. Using random selection to populate the subsets of 9 tweets ensured that each tweet had an equal chance of being in a subset with any other tweet. This method approximates the way social media users might encounter messages, not viewing one message on its own, but viewing several messages during one browsing session. Social media users are likely to encounter misinformation, but because of social media algorithms, a user is unlikely to see the exact same set of tweets as another user.

### Analysis of Tweet Characteristics

#### Tweet Content

In total, 2 independent coders conducted a content analysis of the tweets for persuasive strategies and health concerns. We used prior literature and an initial reading of the tweets to develop a preliminary codebook. After an initial round of coding, the codebook was updated to clarify definitions to increase interrater reliability. For the final round of coding, the interrater agreement for the codes ranged from 0.80 to 1.00 [27], and discrepancies were resolved by a third coder. Persuasive strategies included use of narrative, use of statistics, use of questions, use of metaphors, evocation of negative emotions, invocation of legal or governmental authority, skepticism of pharmaceutical company’s motives, use of youth language and vaccine mandates [28-37]. Health concerns included death, autoimmune diseases, infertility, cervical cancer, HPV infection, fainting, brain inflammation, paralysis or impaired mobility, cancers other than cervical cancer, fibromyalgia, autonomic disorders, vaccine ingredients, hospitalization, “injury,” adverse reactions, and side effects [26].

#### Elicited Concern

The frequency that a tweet elicited concern was calculated as a characteristic for each of the 126 tweets containing misinformation about the HPV vaccine by dividing the number of times a message was selected as concerning by the total number of times it was viewed. For example, if tweet A was in 40 of the subsets of 9, and 35 of the parents who had it in their subset selected tweet A as 1 of the 3 most concerning tweets, the frequency of concern for tweet A would be 87.5%. Similarly, if tweet B was in 36 of the subsets and 6 parents who had it in their subset selected it as most concerning, the concern for tweet B would be 16.7%. If all 126 tweets were equally concerning to parents, the concern calculation for each tweet would be expected to be approximately 33% (3 chances to be selected from a subset of 9).

Beta regression was used to test whether the content of the tweet significantly predicted the frequency that a tweet elicited concern. For the simple beta regression models, we used a Bonferroni adjustment to account for multiple comparisons.

### Ethical Considerations

The study was approved by the University of Pennsylvania institutional review board (851852). Participants provided informed consent before beginning the survey and all data were deidentified. Participants were compensated by Qualtrics according to the terms they agreed upon when joining the panel.

## Results

### Overview

Concern about the misinformation tweets varied widely among parents. The frequency of parents selecting a tweet as most concerning ranged from 2.8% of the time it was viewed to 79.5% of the time it was viewed. Of the 126 misinformation tweets, 18 tweets were selected as most concerning at least 50% of the time they were viewed (see Table 2 for exemplars). All 18 of them evoked negative emotion, and 50% (n=9/18) used youthful descriptors or language denoting care for a younger relative (eg, boy, girl, children, my daughter). A third (n=6/18) of these concerning tweets used narratives or personal stories as evidence against the HPV vaccine and 39% (n=7/18) used numerical or statistical information as evidence. Half of the tweets that elicited concern at least 50% of the time they were viewed mentioned vaccine injury (n=9/18), 44% (n=8/18) mentioned death, and 39% (n=7/18) mentioned side effects or adverse events.

**Table 2:** Exemplars of most concerning tweets.

Tweet text	Percentage of parents indicating the tweet as most concerning, %	Persuasive strategy	Health concern content
13-YEAR OLD BOY GOT PARALYZED FROM NECK DOWN AFTER#Gardasil #HPV #Vaccine https://t.co/qs5Mmxkez7 #VaccineInjury https://t.co/GX8GGA7y1z	79.5	Narrative, negative emotion, youth language	Paralysis, vaccine injury
10% of girls who received the #HPV vaccine ended up in the ER within 42 days - some with reactions & side effects that destroy their lives. @RealCandaceO @eileeniorio @delbigtree @talialikeitis @ChildrensHD @picphysicians @va_shiva @VaccineResist https://t.co/W4npGxkzO8 https://t.co/Ynl5olX18L	73	Statistics, negative emotion, youth language	Hospitalization, side effects
Interesting, had the #HPVvaccine but still got #hpv. So the vaccine is not only ineffective but can also cause #death, cervical cancer and a whole lot of other life changing symptoms. https://t.co/XInr3Gc0Bs	64.5	Narrative, negative emotion	Death, cervical cancer, HPV infection
@POtuS @REALDonaldtRump Merck wants American parents to take their children for an injection that will #KILL 1% of them, permanently disable God knows how many more, to prevent a disease that wouldn't kill a minute fraction of #kill rate of #HPV #vaccine. Your opinion? https://t.co/4pAHnVtUy5	63.4	Statistics, questions, negative emotion, pharmaceutical skepticism, youth language	Death, paralysis or impaired mobility

### Impact of Tweet Content on Parental Concern

Simple and multiple beta regression models were used to test whether specific persuasive strategies and health concern topics present in a tweet significantly predicted the selection of a tweet as most concerning (Table 3). In simple beta regression models with a single strategy or concern as the predictor variable, the use of negative emotional appeals (*β*=.94, *P*<.001) and statistical evidence (*β*=.59, *P*<.001) were the persuasive strategies that significantly increased the likelihood of selection after using Bonferroni correction to adjust for multiple comparisons. The health concern that increased the likelihood of selection was paralysis (*β*=.78, *P*=.002).

In a multiple beta regression model including all the persuasive strategies and health concerns as predictor variables, the overall regression was statistically significant (likelihood ratio *χ*^2^_24_=75.8, *P*<.001). Tweets using negative emotional appeals (*β*=.79, *P*<.001), expressing skepticism about pharmaceutical companies (*β*=.36, *P*=.036), and invoking governmental authority (*β*=.44, *P*=.022) significantly increased parents’ selection of the tweet as most concerning. Hospitalization (*β*=1.00, *P*=.003), paralysis (*β*=.54, *P*=.021), and infertility (*β*=.52, *P*=.035) were the health concerns that increased selection. The use of statistical evidence (*β*=.29, *P*=.064), which was significant in the simple beta regression model, was not significant in the multiple beta regression model.

**Table 3:** Simple and multiple beta regression of persuasive strategies and health concerns on the selection of a tweet as most concerning. Multiple beta regression model (likelihood ratio χ^2^_24_=75.8, *P*<.001). The simple beta regression models account for multiple comparisons using the Bonferroni adjustment. b is the unstandardized regression coefficient. Persuasive strategies and health concerns are binary variables: 0=not present, 1=present. Concern is the frequency that the tweet elicited concern when it was viewed (range 2.8%-79.5%).

	Simple				Multiple		
	b Coefficient (95% CI)	z	*P*>z	b Coefficient (95% CI)		z	*P*>z
Negative emotion	.94^a^ (.50 to 1.37)	4.24	<.001	.79^a^ (.38 to 1.20)		3.75	<.001
Hospitalization	1.14 (.34 to 1.93)	2.79	.005	1.00^a^ (.34 to 1.66)		2.97	.003
Paralysis	.78^a^ (.29 to 1.27)	3.13	.002	.54^a^ (.08 to 1.00)		2.31	.021
Governmental authority	.10 (–.35 to .55)	0.45	.650	.44^a^ (.06 to .81)		2.29	.022
Infertility	.30 (–.29 to .88)	1.00	.316	.52^a^ (.04 to 1.01)		2.10	.035
Pharma skepticism	.36 (–.03 to .76)	1.81	.070	.36^a^ (.02 to .70)		2.10	.036
HPV infection	1.23 (–.17 to 2.64)	1.72	.085	1.19 (–.01 to 2.39)		1.94	.052
Statistics	.59^a^ (.28 to .90)	3.70	<.001	.29 (–.02 to .59)		1.85	.064
Youth language	.28 (.02 to .53)	2.15	.032	.25 (–.02 to .52)		1.80	.072
Questions	–.15 (–.48 to .18)	–0.89	.375	–.24 (–.52 to .05)		–1.62	.104
Side effects	.30 (.02 to .58)	2.06	.039	.21 (–.06 to .47)		1.54	.123
Cervical cancer	.41 (–.03 to .84)	1.82	.069	.29 (–.11 to .68)		1.41	.159
Metaphor	–.03 (–.45 to .39)	–0.13	.898	–.27 (–.64 to .11)		–1.40	.163
Autonomic disorders	.91 (–.07 to 1.88)	1.82	.069	.65 (–.49 to 1.78)		1.11	.265
Death	.39 (.09 to .70)	2.54	.011	.16 (–.13 to .45)		1.10	.271
“Injury”	.02 (–.24 to .28)	0.15	.882	.12 (–.13 to .36)		0.94	.348
Fainting	.21 (–.80 to 1.21)	0.40	.688	.51 (–.60 to 1.63)		0.90	.368
Mandate	–.01 (–.41 to .38)	–0.06	.952	–.15 (–.49 to .20)		–0.84	.400
Auto immune	.35 (–.46 to 1.17)	0.85	.398	.28 (–.40 to .97)		0.81	.416
Other cancers	1.23 (–.17 to 2.64)	1.72	.085	.50 (–1.12 to 2.12)		0.60	.545
Fibromyalgia	–.06 (–1.51 to 1.39)	–0.08	.936	–.45 (–2.02 to 1.11)		–0.57	.568
Ingredients	.14 (–.51 to .78)	0.41	.680	.15 (–.43 to .73)		0.51	.609
Brain inflammation	–.08 (–.68 to .53)	–0.24	.807	–.12 (–.64 to .41)		–0.43	.668
Narrative	.14 (–.14 to .42)	0.98	.329	.00 (–.27 to .28)		0.03	.973
(Constant)	—^b^	—	—	–1.88 (–2.29 to –1.48)		–9.12	<.001

^a^*P*<.05.

^b^Not applicable.

## Discussion

### Principal Findings

Parental concern about the different misinformation tweets varied widely, with some misinformation being selected as most concerning just 2.8% of the time it was viewed and other misinformation being selected 79.5% of the time it was viewed. Multiple beta regression analyses found that the misinformation content that parents of 7- to 10-year-olds found most concerning were mentions of hospitalization (b=1.00), paralysis (b=.54), and infertility (b=.52), and the tweets using the persuasive strategies of negative emotional appeals (b=.79), expressing pharmaceutical company skepticism (b=.36), and invoking governmental authority (b=.44) were most concerning to these parents.

Using strong emotions and direct language are common user strategies on social media [38]. Our study found that parents were more likely to select tweets that used negative emotions and statistical evidence as most concerning with regard to intent to vaccinate their child. This aligns with prior research; parents exposed to negative vaccine information are less likely to vaccinate their child, as negative tweets are memorable and personal [22,39]. The use of negative emotions and statistics are effective strategies to increase concern about a health topic for health campaigns. The use of narratives and statistical evidence was effective in increasing risk perception associated with the HPV vaccine in previous research [40].

Tweets expressing skepticism about pharmaceutical companies and invoking governmental authority were more likely to elicit concern among parents compared to tweets that did not. Generally, negative vaccine sentiment in digital articles centered around distrust in institutions that govern personal health and skepticism toward organizations that deliver scientific evidence, including pharmaceutical companies [41]. Distrust in pharmaceutical companies, related to conspiracy theories, has been common on social media. In antivaccination “memes” collected in a study, one of the main themes present was conspiracy theories about government and pharmaceutical companies, which were more prevalent in antivaccination content compared to pro-vaccination content [42].

Vaccine safety concerns are one of the top reasons why parents and children refuse HPV vaccination, regardless of gender [43]. Antivaccine users describe serious but scientifically unconfirmed injuries, safety issues, and side effects, including death, to connect to readers, often describing vaccines as harmful to health [38]. Messages about HPV vaccine harm are more associated with vaccination behavior compared to messages on HPV vaccine preventable diseases [44]. For specific safety concerns in our study, tweets containing content on hospitalization, paralysis, and infertility elicited concern more frequently than tweets that did not, whereas tweets about other safety concerns, ingredients, and side effects were topics that did not predict the elicitation of concern.

### Prevalent Misinformation and Most Concerning Misinformation

In addition to being concerning, the topics of safety and pharmaceutical skepticism are also some of the most prevalent topics about HPV on social media. For example, concern for the vaccine being part of a conspiracy between the government and pharmaceutical companies was one of the top 3 frequent comments from both pro- and anti-HPV vaccine YouTube videos [45]. In another study, the top 3 categories of HPV vaccine-hesitant tweets were vaccine safety, vaccine effectiveness, and mistrust in government and vaccine mandates [46]. Antivaccine content is more popular on social media than pro-vaccine content, thus showcasing a possible relationship between prevalence on social media and antivaccine content [38]. Posts about HPV vaccine mandates are prevalent on social media, but they were not predictive of being selected as concerning in our study.

Some of the topics identified as concerning in our study vary from what providers report as concerns that they hear about from parents. Besides vaccine safety, providers report hearing concerns from parents about their children engaging in sexual activity as a result of getting the vaccine [47-49]. In a separate study, parents reported other concerns as reasons for refusal and delay, including that their child was not sexually active, potential health problems caused by the vaccine, and their children not needing to be vaccinated against HPV; parents who had previously refused or delayed HPV vaccine uptake reported that they did so because they required more information on the vaccine [50]. Future studies should further assess concerns about sexual activity compared to the other health topics. Providers highlighted knowledge deficits and misinformation exposure as barriers to care and believe that prevaccination education on side effects and benefits must be addressed to promote HPV vaccine uptake [48,51].

### Limitations

This study is among the first to use social media messages containing HPV vaccine misinformation obtained directly from Twitter as stimuli. While this design allows us to measure reactions to actual misinformation being circulated on Twitter [26], the tradeoff is that some health concerns are more prevalent in the sample of 126 tweets than others. Qualtrics panels recruited a convenience sample of participants who were whiter and more educated than the United States [52], and the results may not be generalizable to a wider population.

### Conclusions

Health misinformation exposure is highly prevalent on social media [24], and viewing vaccine misinformation may directly influence vaccination opinions, contribute to downstream vaccine hesitancy, and ultimately lead to refusal or delay of vaccination [12,14-16]. It would be impossible to target and combat all the topics and categories of misinformation about the HPV vaccine circulated on social media, but not every piece of misinformation influences beliefs and vaccine hesitancy [53], and as this study found, parents do not find all misleading information equally concerning. This study provides valuable information to help understand the types of misinformation on social media that parents find concerning regarding vaccine behavior and can inform the development of new and innovative strategies to effectively combat the influence of misinformation on HPV vaccine uptake. Prior research has been done to begin this process, showcasing the types of misinformation shown on social media [26,38]. Identifying concerns to target is essential, as targeting misinformation that is prevalent but not concerning could result in combating misinformation without impacting vaccination behavior. This research helps to identify the characteristics of HPV vaccine misinformation on social media that parents find most concerning. Future research can test strategies to address these concerns and target the effects of misinformation.

## Abbreviations

HPV: human papillomavirus

## Data Availability

The datasets generated and analyzed during this study are not publicly available due to restrictions form the institutional review board but are available from the corresponding author on reasonable request.
